# Effects of Threat Conditioning on the Negative Valanced Systems and Cognitive Systems

**DOI:** 10.1038/s41598-018-29603-3

**Published:** 2018-07-25

**Authors:** Rodrigo S. Fernández, Soledad Picco, Fernando Messore, María E. Pedreira

**Affiliations:** Universidad de Buenos Aires, Facultad de Ciencias Exactas y Naturales, Departamento de Fisiologia, Biologia Molecular y Celular, CONICET-Universidad de Buenos Aires, Instituto de Fisiología, Biología Molecular y Neurociencias (IFIBYNE), Buenos Aires, Argentina

## Abstract

Threat conditioning is held as a model of anxiety disorders. However, this approach is focused on implicit responses evaluated in a single day. Here, we evaluated negative-valence, positive-valence and cognitive-systems in order to evaluate the extent to which threat conditioning models anxiety disorders. Subjects underwent threat conditioning and five-minutes (Short-term evaluation) or 48 hs (Long-term evaluation) later, both groups performed several tasks targeting cognitive-systems and valenced-systems. In the short-term evaluation, successful conditioning maintained state-anxiety and increased the aversiveness representation of the CS+ and the valuation for negative events. Reaction-times for the CS+ were faster, reflecting an attentional bias toward threat. In the long-term evaluation, participants represented the CS+ as more aversive and generalized to all stimuli. Reaction-times showed a more restricted attentional bias. Threat conditioning alters the negative-valence systems and creates a cognitive bias, which is transformed by memory consolidation, suggesting that this protocol could be a useful resource to understand the deficits associated with anxiety disorders.

## Introduction

Anxiety and fear-related disorders as a group are the most prevalent mental illness^1,2^. Individuals with anxiety disorders are excessively anxious, fearful and avoidant of a large range of internal or external threats. At the behavioral level, fear and anxiety frequently overlap; however, they can be differentiated by considering the level of uncertainty regarding the probability, timing or nature of future danger^3–5^. Fear refers to a defensive motivational response to an actual (acute) or immediate threat^6^. In contrast, anxiety is defined as a persistent and generalized defensive response to a potential, unpredictable or uncertain threat^3,7,8^. Typical anxiety symptoms are physiological (i.e., muscle tension, palpitations, dizziness, nausea), cognitive (i.e., fear of losing control, hypervigilance, worry), behavioral (i.e., avoidance, freezing) and emotional (i.e., arousal, fearfulness, impatience).

From Pavlov’s “experimental neurosis”^9^ to the modern neuroscientific theories^10,11^, it is clear that learning, and more specifically, Pavlovian threat (fear) conditioning, plays a critical role in the pathogenesis and maintenance of anxiety disorders^11,12^. Ultimately, anxiety disorders are not acquired in an instant and actually develop gradually. However, threat conditioning in animals and healthy populations is held as a model of anxiety- and fear-related disorders. As a laboratory model, threat conditioning, conceptualized as an aversive-implicit memory, has allowed researchers to gain tremendous knowledge about the neurobiological basis of anxiety disorders and to develop novel treatments^13,14^. However, considering that anxiety disorders engage physiological, emotional and cognitive changes, this approach reduces the modeled disorder to an acquired associative response at physiological (i.e., heartbeat interval, electrodermal activity, etc.) and declarative (i.e., contingency awareness) levels. As a result, this research bias neglects fundamental aspects of threat processing and anxiety disorders such as valenced (positive/negative), cognitive or regulatory domains.

On the other hand, the current diagnosis system is suffering a shift from a categorical classification (DSM V) to a dimensional one including a constellation of factors. Consequently, the Research Domain Criteria (RDoc)^15^ proposed a framework to study mental illness using a dimensional matrix that considers different domains (positive, negative, cognitive, regulatory or social systems) and units of analysis (from genes to behavior). In this regard, highly anxious individuals could be thought as having alterations in negative-valence and cognitive system processing. Negative valence systems are involved in the anticipation, maintenance and processing of aversive situations (acute/potential threat, loss, etc.), and the cognitive systems are a set of interrelated cognitive processes such as attention, cognitive control, perception and language.

Thus, a classical threat conditioning paradigm targets only the acute threat construct of the negative system domain. Highly anxious individuals show an orchestrated response within various systems^16^, which alters the inner experience of the subject. This idea is also supported by the diversity of cognitive, emotional and behavioral symptoms in anxiety disorders. Moreover, if one considers that anxiety functions to anticipate, prepare and respond to actual and potential threats^3^, it makes sense that it does not rely on only one processing system (negative valence system, i.e., physiological reactivity to cues) and that several cognitive processes might be involved. For example, highly anxious individuals show a faster detection of negative material (attentional bias towards threat), a stronger response to aversive cues (heightened reactivity to threat), recall more threat-related information (memory bias) and overestimate the probability and severity of negative events (exaggerated threat-value calculations)^3,7^, all of which are not generally targeted by current threat conditioning paradigms in normal/healthy subjects. Generally, learning and memory as primary brain processes are able to control, bias or affect several cognitive and emotional processes^11,13^. Previous studies on threat conditioning have focused on single dimensions such as attentional and evaluative components, showing that in the short term, fear acquisition can produce an attentional bias and an increment of the aversive appraisal to fear-related cues^17,18^. However, it is important to note that another fundamental, missing aspect is whether an acquired fear memory is able to maintain its effects on cognitive and valenced systems with the passage of time. Extensive evidence supports the idea that once acquired, a short-term memory passes through a stabilization phase, called memory consolidation, in order to persist^19^. This neurobiological process implies changes in the content, strength and “resolution” of the stored representation such as forgetting, generalization and gist memory^20–24^.

**Figure 1:**
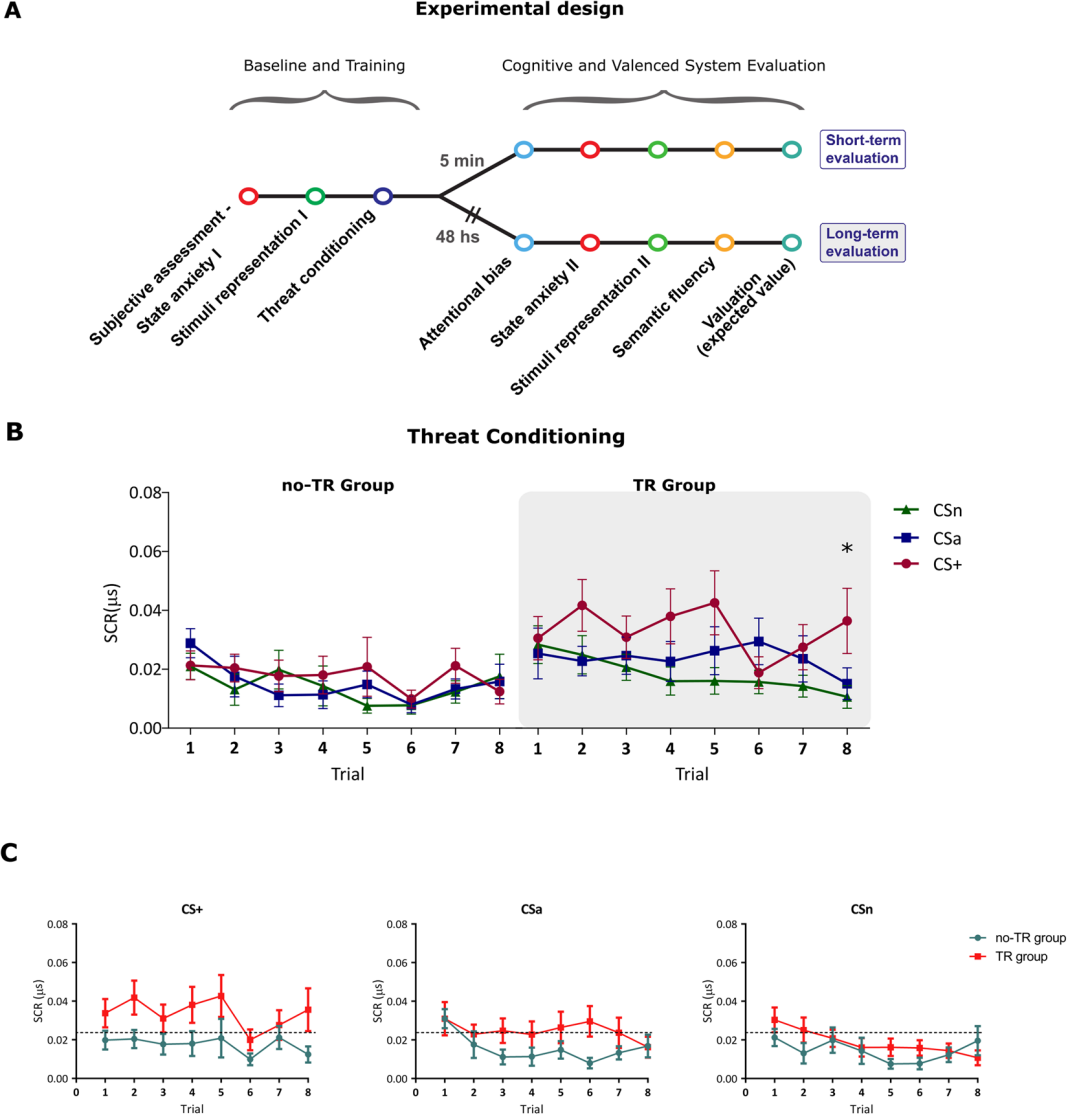
Experimental timeline and threat conditioning. (**A**) Experimental design. Left *panel*. After arriving, the subjects were first assessed (BAI, STAI-T), completed the first part of the aversiveness rating task (stimuli representation) and then underwent threat conditioning or a control task (no-US). *Right Panel*. Five minutes (Short-term evaluation) or 48 hs (Long-term evaluation) later, the participants completed several tasks targeting the negative-valence (state anxiety, stimuli representation, negative valuation), positive-valence (positive valuation) and cognitive systems (attentional bias and semantic fluency). (**B**) Threat conditioning. Subjects in the TR groups successfully acquired the aversive memory while those in the control task (no-TR group) did not. (**C**) Mean SCR (μS) for CS+, CSa and CSn for TR and no-TR groups in the Short-term evaluation and the Long-term evaluation. Mean SCR (μS) ± SEM, *P < 0.05.

Here, inspired by the RDoc matrix and their conceptual systems, we aimed to study how a given experience (memory) could affect the complexity of different valenced and cognitive systems and to determine their persistence (consolidation) in a normal population. To reach this goal, subjects first were assessed and then underwent threat conditioning or an equivalent control task (without an US). Five minutes (short-term evaluation groups) or 48 hs (long-term evaluation groups) later, subjects performed different tasks (Fig. 1A) systematically targeting negative-valence (state anxiety, stimuli representation and valuation), positive-valence (valuation) and cognitive systems (attentional bias and semantic fluency). In other words, in this study, we asked whether threat conditioning is capable of producing and maintaining a change in valenced (positive/negative) and cognitive systems capturing the deficits associated with anxiety- and fear-related disorders.

## Results

### Subjective assessment and threat conditioning

Seventy participants completed the subjective assessment and were randomly assigned to a threat-training (TR) or no-training (no-TR) group. Those in the TR group underwent threat conditioning, and subjects in the no-TR group performed the same task in the total absence of the US. In the Short-term evaluation (n_TR_ group = 19, n_no-TR_ group = 18), five minutes after conditioning, both the trained and not-trained groups were evaluated for negative-valence, positive-valence and cognitive systems using several different tasks (Fig. 1A, *see below*). In the Long-term evaluation (n_TR_ group = 17, n_no-TR_ group = 16), two other independent trained and not-trained groups were evaluated 48 hs after threat conditioning, using the same tasks. To obtain an assessment of subjects’ trait anxiety and the presence of physiological symptoms, the State-Trait Anxiety Inventory (STAI-S and STAI-T^16^) and the Beck Anxiety Inventory (BAI^17^) questionnaires were used. Participants in the Trained (TR) and not-Trained (noTR) groups did not differ in self-reported trait anxiety (STAI-T, TR group *mean:* 32.61 ± 1.07 and noTR *mean:* 32.22 ± 1.31; *t*_64_ = 0.223) or anxiety symptomatology (BAI; TR group *mean:* 10.24 ± 1.21 and noTR *mean:* 9.27 ± 0.98; *t*_62_ = 0.492). Then, three different male-face pictures (CS1, CS2, and CS3) serve as the conditioned stimulus (CS). Two of them (CS1 and CS2) expressed anger on their faces, and the other one (CS3) was neutral. In the trained groups, either CS1 or CS2 was associated with the unconditioned stimulus (US) in 75% of the trials in a counterbalanced manner. Hereinafter, we will call the aversive reinforced stimulus “CS+”, the unreinforced aversive stimulus “CSa” and the neutral stimulus “CSn”. The no-training group received the same instruction and performed the same task in the absence of the tone-US.

We analyzed threat conditioning (SCR) using a mixed repeated-measure ANOVA with Group as between-subjects factor and Stimulus (CS+, CSa and CSn) and Trial (1–8) as within-subjects factors. Successful threat acquisition was found in the TR group (Fig. 1B; mixed repeated-measures ANOVA, Group × Stimulus × Trial Interaction: F_2.128_ = 3.21, P = 0.03, η_p_^2^ = 0.48). Moreover, there was a differential increase in skin conductance response (SCR) amplitudes for CS+ relative to CSa and CSn from the first to the last trials in the TR group but not in the no-TR group (Simple effects last trial P_(CS+ vs all cs)_ < 0.005). At this point, both groups were then randomly assigned to a short-term (5 min) or long-term evaluation (48 hs later) of valenced (negative/positive) and cognitive systems.

The overall effect of threat conditioning on the negative-valence, positive-valence and cognitive systems was analyzed by means of a 2 (Group: trained vs not-trained) × 2 (Time: short-term evaluation vs long-term evaluation) MANOVA followed by separate Two-way ANOVA’s for each dependent variable. The two-way MANOVA revealed a robust effect of training (Group: Wilks’ λ = 0.475, F_9.00_ = 7.00, P < 0.001, η_p_^2^ = 0.535), Time (Wilks’ λ = 0.690, F_9.00_ = 2.93, P < 0.001, η_p_^2^ = 0.329), as well an interaction (Group × Time: Wilks’ λ = 0.687, F_9.00_ = 2.890, P < 0.001, η_p_^2^ = 0.313) on the negative-valence, positive-valence and cognitive systems. In order to disentangle these effects, separate 2 (Group: trained vs not-trained) × 2 (Time: Short-term vs Long-term evaluation) ANOVA’s (Group × Time) were performed on each dependent variable.

### Threat conditioning effects on the negative valence and positive valence systems

#### State Anxiety

**Figure 2:**
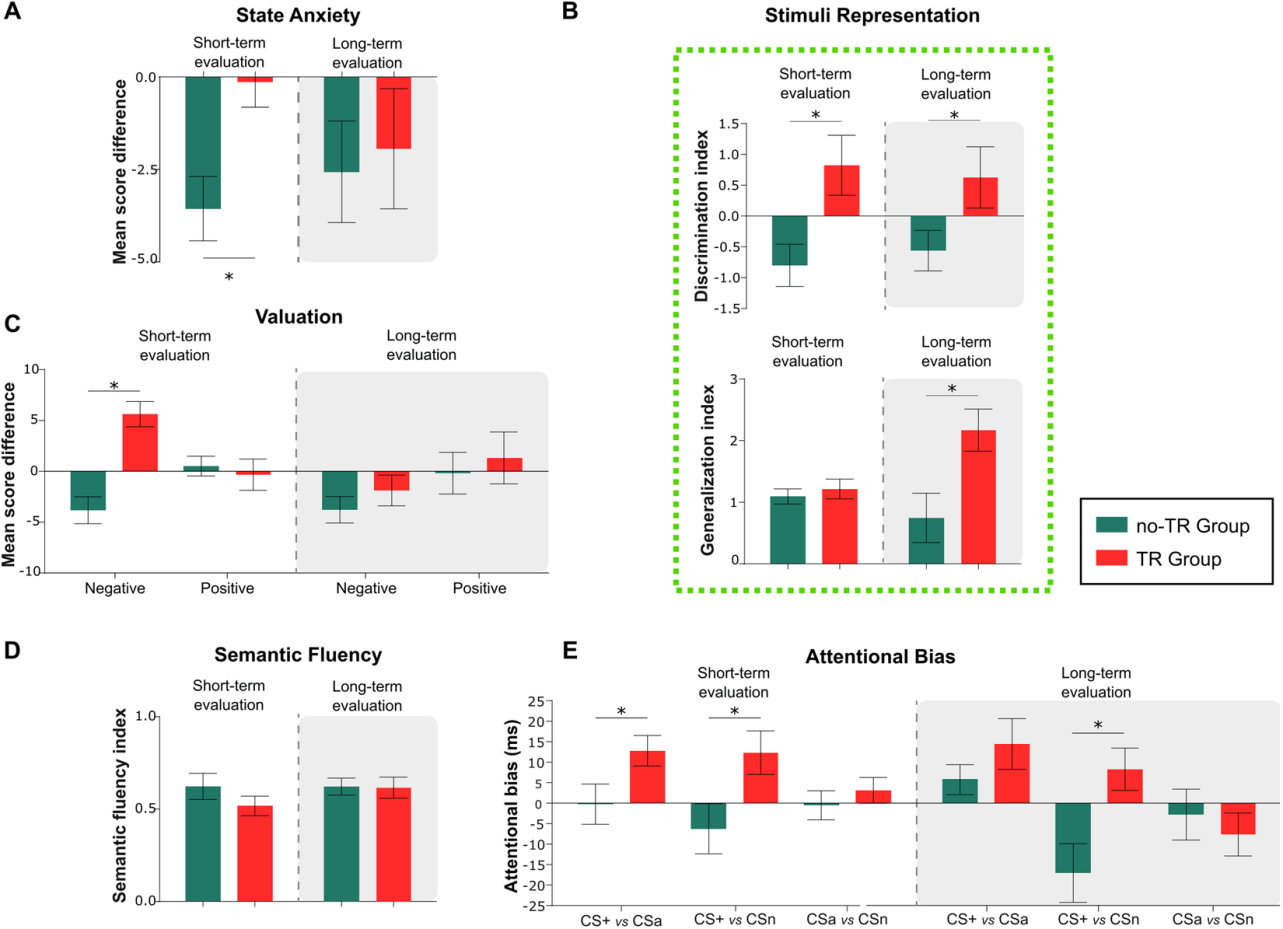
Threat conditioning affects negative-valence and cognitive systems after memory acquisition (Short-term evaluation) and memory consolidation (Long-term evaluation). (**A**) State anxiety: mean difference in STAI scores before and after training. (**B**) Stimuli representation (aversiveness): *Upper panel:* Discrimination index mean difference for CS+ and CSa ratings; *Lower panel:* Generalization index: relation between mean ratings for aversive pictures and neutral pictures (not seen during training). (**C**) Valuation: Subjects assigned negative and positive expected values (probability x cost in a 0–8 scale) for hypothetical events regarding CS+ and CSa. (**D**) Semantic fluency: Relation between the number of words generated for the neutral and negative category. Blue bars represent no-TR groups, and red bars represent TR groups (**E**) Attentional bias (dot probe): Difference in ms between incongruent and congruent trials for 3 types of stimuli compounds (CS+ vs CSa, CS+ vs CSn and CSa vs CSn). *P < 0.05.

Current anxiety or perceived distress levels were measured by the mean difference in STAI-S scores between post- minus pre-threat conditioning for each group (Fig. 1A). A main effect of training was found on state anxiety (Two-way ANOVA, Group factor: F_1.65_ = 4.69, P < 0.005, η_p_^2^ = 0.66) but not for evaluation Time (F_1.65_ = 0.808, P > 0.05) or interaction (F_1.65_ = 2.33, P > 0.05). In the short-term evaluation groups, participants in the TR group maintained a similar perceived distress level (STAI-S score difference) before and after training, relative to the no-TR group (Fig. 2A, follow up pairwise comparison P < 0.01). As expected, the long-term evaluation, found no differences between TR and no-TR groups (STAI-S; P > 0.05). This result is reasonable, considering the mild “threat” level during threat conditioning, the unspecific arousal measurement of STAI-S and the 48 hs between the first and second measurements.

#### Stimuli Representation (aversiveness)

Before (pre) and after (post) threat conditioning (Fig. 1A), subjects were instructed to rank on a 0 to 8 scale how aversive or unpleasant were 10 pictures of male faces (5 angry and 5 neutral, including the CS+, CSa and CSn pictures) presented randomly on the monitor. With the mean scores, we calculated a stimuli representation *discrimination index* (CS+ vs CSa) and a *generalization index* (all aversive pictures vs all neutral pictures) for each group. In both the short-term and long-term evaluations, we found a significant effect of threat conditioning training on discrimination index (Two-way ANOVA, Group factor: F_1.65_ = 11.128, P < 0.001, η_p_^2^ = 0.15). Evaluation Time (F_1.65_ = 0.140, P > 0.05) or interaction (F_1.65_ = 0.118, P > 0.05) were not significant. Subjects in the TR groups rated the CS+ as more aversive and unpleasant than the CSa (*Discrimination Index* – Fig. 2B upper panel: follow up pairwise comparison: Short-term evaluation P < 0.01 and Long-term evaluation P < 0.05). Regarding the *Generalization index*, the two-way ANOVA revealed only a main effect of training (Group: F_1.65_ = 10.515, P < 0.001, η_p_^2^ = 0.15; Time: F_1.65_ = 0.99, P > 0.05) and a significant Group × Time interaction (F_1.65_ = 9.287, P < 0.01, η_p_^2^ = 0.12) revealing a generalization effect after consolidation (Long-term evaluation). Notably, the “acquired aversion” for the CS+ (TR groups) in the short-term evaluation did not generalize to the other negative stimuli (*Generalization Index* – Fig. 2B Lower panel: simple effects: P > 0.05). However, 48 hs after threat conditioning (once consolidation ended), participants in the TR group showed a generalization effect and rated all the negative stimuli presented as more aversive relative to the neutral stimuli (Long-term evaluation, *Generalization Index* – Fig. 2B: simple effects: P < 0.005).

#### Valuation

We designed a task in order to assess the subjective expected value assigned to the aversive CSs (CS+ and CSa) in imagined positive or negative scenarios. Following threat conditioning, the subjects were presented randomly on the computer with 24 positive and 24 negative situations involving either CS and its pictures as a “fictional character” (12 valenced situations for CS+ and CSa). For each positive and negative scenario, the subjects first responded on a 0 to 8 scale how likely was the hypothetical event and then how good/bad it would be for them, using the same scale. We then calculated the expected value (subject-assigned mean probability multiplied by its mean negative/positive magnitude) for CS+ and CSa in positive and negative situations for each group (trained and not-trained). Figure 2C displays the mean score difference for the estimated positive/negative expected values for the Short-term and the Long-term evaluation. A main effect of training and evaluation time was found for the negative scenarios (Two-way ANOVA, Group: F_1.65_ = 19.230, P < 0.001, η_p_^2^ = 0.30; Time: F_1.65_ = 7.257, P < 0.01 η_p_^2^ = 0.10) and their interaction (F_1.65_ = 5.696, P < 0.05, η_p_^2^ = 0.10). In the Short-term evaluation, the TR group compared to the no-TR group rated as more likely and adverse the negative scenarios involving the CS+ relative to the CSa (simple effects P < 0.001). We found no evidence of this effect for the positive scenarios (F_1.65_ = 0.016, P > 0.05). Regarding the long-term evaluation, we found no difference between TR and no-TR groups in either negative or positive scenarios (simple effects negative: P > 0.05; positive: P > 0.05), revealing that the higher negative expected value did not persist over time or its generalized in negative scenarios between the aversive CS’s.

In summary, these results show that threat conditioning (TR group) specifically affects the negative valence systems and not the positive valence systems (Positive expected value). Moreover, threat training has more impact at short-term evaluation than at long-term evaluation, suggesting that some effects on the negative valence systems underwent a generalization process or did not survive the memory consolidation process.

### Threat conditioning effects on the cognitive systems

#### Semantic fluency

Subjects were asked to generate as many as possible words aloud in 60 sec belonging to a neutral category (words related to a supermarket) and an aversive category (words related to fear and negative emotions)^25^. The mean number of words generated by subjects in each category was used to estimate a fluency index (Aversive_category_/Neutral_category_) for each group. For the short-term and long-term evaluations, both the TR and no-TR groups generated roughly the same number of words (during 1 min) in the neutral or negative category (Fig. 2D; Two-way ANOVA Group: F_1.65_ = 1.125, P > 0.05; Time: F_1.65_ = 1.140, P > 0.05; Interaction: F_1.65_ = 0.685, P > 0.05).

#### Attentional bias

Subjects performed a pictorial dot probe task^26^ to evaluate the existence of an acquired attentional bias toward threat. Each trial began with a fixation cross in the center of the screen for 500 ms, followed by the simultaneous display of two CSs (left/right) for 500 ms. After the brief stimulus presentation, the CS picture disappeared, and a small white probe appeared in the previous location (left/right) occupied by one of the CSs for 1000 ms (Fig. 1A). Using an external keypad with two buttons, participants were asked to press a button as fast as they could, according to where they detected the white probe (left/right). Participants had 2000 ms to respond in each trial. Here, we used 4 types of stimulus compounds: (1) CS+ vs CSa; (2) CS+ vs CSn; (3) CSa vs CSn and (4) two neutral faces that had not appeared before in the experiment (*filler* trials). An attentional bias is inferred from faster reaction times (RTs) on trials where the probe is presented at the previous location of the threatening cue (*congruent trials*) compared to those where the target is located at the previous location of the non-threatening cue (*incongruent trials*). In our protocol, we considered *congruent trials* to be when the probe appeared at the CS+ location (stimulus compounds 1 and 2) or CSa (stimulus compound 3) and *incongruent trials* to be the remaining possibilities (probe CSa or CSn on stimulus compounds 1 and 2 and CSn on compound 3). The mean RT from the dot probe task was estimated for each group *per* condition taking the difference between incongruent and congruent trials. A two-way ANOVA revealed a significant effect of threat conditioning on reaction times for CS+ vs *CSa* (Group: F_1.65_ = 4.700, P < 0.05, η_p_^2^ = 0.10) and CS+ vs *CSn* (Group: F_1.65_ = 14.258, P < 0.001, η_p_^2^ = 0.18) but not for evaluation time (F_1.65_ = 2.247, P > 0.05 and F_1.65_ = 1.091, P > 0.05, respectively) or their interaction (F_1.65_ = 0.718, P > 0.05 and F_1.65_ = 0.700, P > 0.05, respectively). In the Short-term evaluation, we found a significant acquired attentional bias toward threat (dot-probe task) in the TR group compared to the no-TR group (Fig. 2E left). In the TR group, RT and probe detection were faster for the CS+ relative to the CSa (CS+ vs *CSa*: pairwise comparisons P < 0.05) or CS-n (CS+ vs *CSn:* pairwise comparisons P < 0.01). Figure 2E shows the results of the dot-probe task in the Long-term evaluation. Here, we found a persistent attentional bias toward threat, specifically in the TR group, only when the CS+ was confronted with the CS-n (Fig. 2E right, CS+ vs *CSn:* P < 0.01) but not with Csa (CS+ vs *CSa*, P > 0.05). Finally, the RTs for not-trained aversive CS (CSa) against the neutral CS (CSn) were similar in the TR and no-TR groups in both the Short-term evaluation and the Long-term evaluation (Two-way ANOVA, Group: F_1.65_ = 0.005, P > 0.05; Time: F_1.65_ = 1.615, P > 0.05; Interaction: F_1.65_ = 0.589, P > 0.05).

These results suggest that not all cognitive systems are affected by threat conditioning. We showed that an attentional bias towards threat could be acquired and maintained after memory consolidation (Long-term evaluation). However, the semantic component remained unaffected.

### Group and system clustering

**Figure 3:**
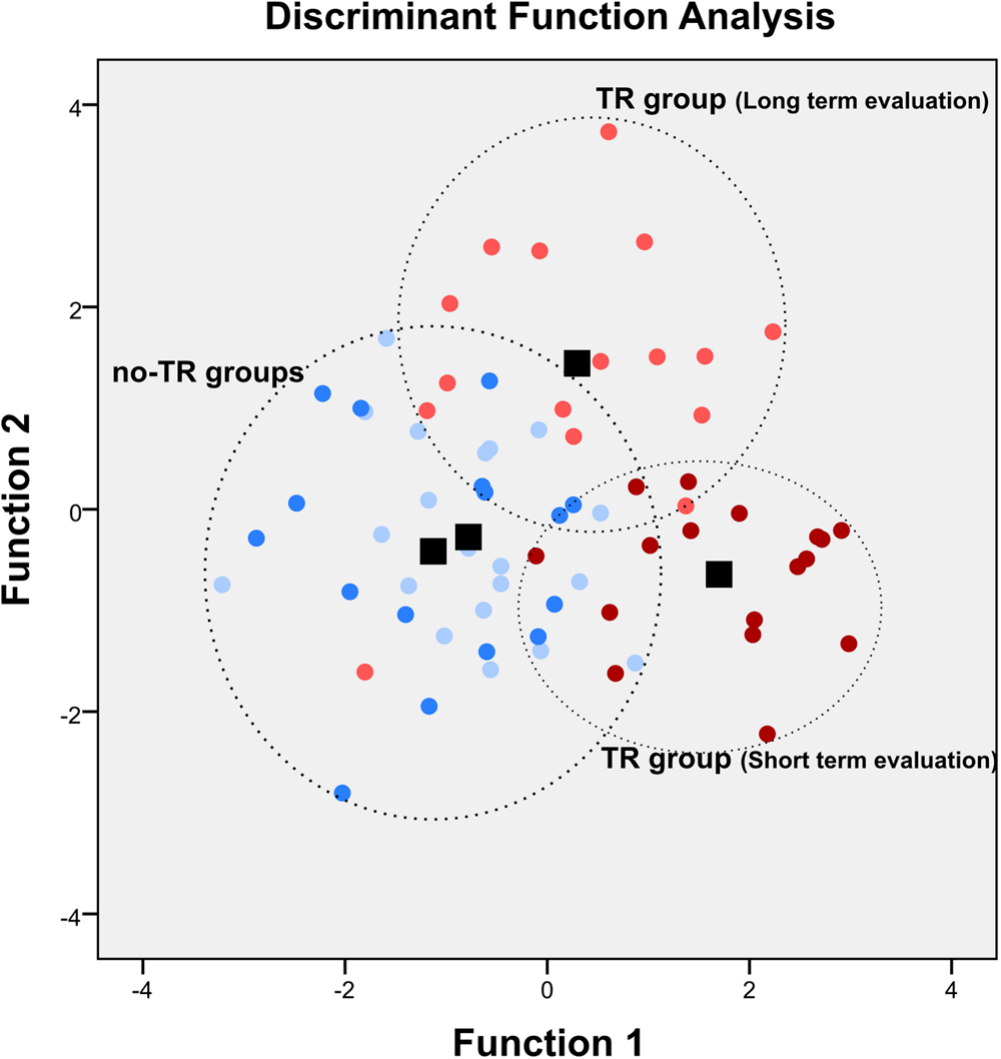
Negative-valence and cognitive systems accurately predict individuals’ acquired threat value of cues through threat conditioning. Functions at group centroids: *Function 1* maximally separates trained (light and dark red) from not-trained (light and dark blue) groups and *Function 2* differentiates groups at short- (dark red) or long-term (light red) evaluation. Black squares stand for group centroids.

To verify if the negative-valence, positive-valence and cognitive systems are able to discriminate groups, we performed a discriminant analysis using the entire but without considering threat conditioning values. The discriminant analysis revealed three functions, two of which were significant (P < 0.001, Fig. 3): the first explained 60% of the variance, canonical *R*^2^ = 0.60, and the second clarified 33% of the variance, canonical *R*^2^ = 0.42. In combination, the discriminant functions significantly differentiated between TR and no-TR groups (*First function*: Wilks’ λ = 0.23, χ^2^_27_ = 92.33, P < 0.001; *Second function*: Wilks’ λ = 0.51, χ^2^_16_ = 41,73, P < 0.001). Moreover, we found a difference in the contribution of each measure to group separation. In the first function, correlations between outcomes and the discriminant function (structure matrix) revealed a fair correlation with negative expected value (*r* = 0.77) and attentional bias (*r* = 0.50) and a poor correlation with positive expected value (*r* = 0.03) and stimuli aversiveness (generalization index r = 0.06). In the second function, the best correlation was found for the stimuli aversiveness (generalization index *r* = 0.87 and discrimination index *r* = 0.60) and here, semantic fluency had the worst association (*r* = 0.04). The classification results of this model accurately predicted group membership for 75.5% of the cases. This analysis indicates that, leaving threat conditioning aside, subjects could be correctly classified according to their performance in the negative-valence, positive-valence and cognitive systems.

We next further investigated the cognitive and valenced systems by means of a principal component analysis (PCA) to assess its viability as constructs in our experimental design. A PCA was conducted on the 4 groups, using the significant measures from the Two-way MANOVA with varimax rotation. Sample adequacy and appropriate correlation between items were verified by the KMO test = 0.55 (all individual values were above >0.5) and Bartlett test of sphericity χ^2^_15_ = 26.50, P = 0.03 respectively. Using eigenvalues >1, we found two factors that explain 59.3% of the variance. Table S2 (Supplemental Material) shows the factor loading after rotation and the components in each factor. These results suggest, as expected, that the cognitive systems (attentional bias) and the negative valence system (discrimination index, STAI-S and negative expected value) are differently affected.

## Discussion

The current findings provide evidence that an experience can change negative valenced systems and create a specific cognitive bias in a manner congruent with the valence of the acquired memory. Hence, these changes in cognitive and negative-valence systems are not transient or a byproduct of short-term memory facilitation. The acquired behavioral profile is maintained or transformed with the passage of time. Here, we showed that standard threat conditioning alters the negative valence systems and creates a cognitive bias. In the Short-term evaluation we revealed that, 5 min after successful conditioning (TR group), (1) subject’s state-anxiety and aversiveness representation of the CS+ (discrimination index) increased, along the estimated probability and cost for hypothetical negative events (valuation) and (2) RTs for the CS+ when confronted with the CSa or CSn were faster, reflecting an attentional bias toward threat. In contrast, in the Long-term evaluation, which was conducted after memory consolidation (48 hs after threat conditioning), we found a different system profile, which revealed the following: (1) Participants represented the CS+ as more aversive than the unreinforced CSa, although here, all the aversive pictures were also perceived as being more negative (discrimination and generalization index); (2) RTs were only faster for the CS+ relative to the CSn, suggesting a more restricted attentional bias compared to groups evaluated 5 min after conditioning; (3) State-anxiety and negative expected values in the TR group did not differ from those in the no-TR group. These results suggest that after memory consolidation, a generalization process between the trained cue (CS+) and the others occurred. In the Short-term evaluation and the Long-term evaluation, semantic fluency and positive valuation were unaffected, indicating that threat conditioning as an aversive memory did not affect all the cognitive or valence systems. Additionally, the discriminant analysis revealed that the experimental tasks are reliable predictors of group membership, and the PCA confirmed the existence of only two factors in our data (cognitive systems and negative valence system).

At this point, our proposal is to analyze present results in the framework of the consolidation process. This process and the later transformations of the memory traces imply that memories are dynamic rather than static, and they do not represent an instant photograph of a specific event. Here, a consolidated fear memory maintains a specific bias in cognitive and valence systems. As was postulated, memories not only are integrated into the existing network but also suffer a time-dependent transformation. As a consequence, the strength and specific features (content) of the trace vary with the passage of time. In this sense, the recently acquired threat conditioning constitutes a trace with moderate strength, high level of precision (details) and low level of integration with previous networks. However, with the passage of time and memory consolidation, the stored representation of threat memory is changed. Thus, forgetting of some aspects, as an adaptative process, is involved in preventing memory overfitting and allowing memory generalization^22^. As a consequence, the consolidated threat-memory is now a trace with moderate-to-low strength, moderate level of precision (details) and more interconnected with previous networks. We can use the same line of thinking for the construction of the memories associated with anxiety disorders to understand their effects on cognitive systems and the specific bias to a more generalized type of stimuli. These memories change the relevance of each feature and the way of processing actual information every moment. Our results are in line with this concept, as the results observed 48 hours after training are the outcome of such transformation.

Over the past decades, the study of threat or “fear” conditioning as a model of anxiety disorders has focused on peripheral sympathetic responses to trained cues (implicit memory). However, it become clear that anxiety disorders are far more than an associative response^27,28^. Several models have assigned a central role to anticipatory responses to threat uncertainty as a transdiagnostic feature across anxiety disorders^3,29^. These responses involve a differential processing of cognitive and negative-valence systems at physiological, behavioral and cognitive levels. For example, highly anxious individuals show an exaggerated threat appraisal of the probability and cost of potential or rare negative events (disrupted valuation). Moreover, the intensity of the response to predictable or unpredictable cues is increased (heightened reactivity to threat uncertainty). Finally, individuals with anxiety disorders exhibit a marked attentional bias toward threat and negative events (increased threat attention and hypervigilance)^26,30^. Our results are in line with these ideas, showing that threat training in a healthy population is able to generate similar anticipatory responses to threats such as those encountered in anxiety disorders. One possible limitation of our experimental design was that the positive valence domain was only represented by a subset of items in a single task. Another limitation comes from the training strength of the threat conditioning. Our protocol generated a memory of “moderate” strength which differs in intensity from the exaggerated fear commonly observed in anxiety disorders. It could be speculated that, differences in training strength could produce different profiles on the negative valence and cognitive systems such as a more detailed memory at the expense of generalization after stronger trainings (i.e. negative valuation or attentional bias may survive memory consolidation). However, despite such limitations, this study contributes a novel approach in the context of the RDoC domains. Future studies, including tasks associated with the positive valence system in healthy subjects and a comparison with subclinical profiles of anxiety, will open new avenues to deeply understand the origin and maintenance of these pathologies.

The RDoc proposed a matrix composed of several systems with the objective of generating a new framework to study and treat mental disorders. In this regard, we believe that acute threat conditioning followed by tasks targeting cognitive and valenced systems of the RDoc matrix could be a useful resource in normal populations to better understand the complexity of mental illness.

## Methods

### Participants

A total of 70 undergraduate and graduate youths (40 females and 30 males) from Buenos Aires University (Argentina) with a mean age of 23.7 ± 0.5 years participated in the current study. Five other subjects were excluded from the analysis based on the inclusion criterion (*see the subjective assessment and threat conditioning subsections*). Before the experiments, participants signed a written informed consent form approved by the Ethics Committee of the Review Board of the *Sociedad Argentina de Investigación Clínica* in accordance with the Declaration of Helsinki.

### Subjective assessment

Only subjects with low to moderate anxiety (STAI-T <45 and BAI < 29) were included in the analysis because highly anxious individuals show increased physiological reactivity (i.e., electrodermal activity, startle reflex) during threat conditioning and an altered learning rate^31^.

### Threat conditioning

Three fear-relevant stimuli served as the conditioned stimulus (CS)^32^. The pictures were taken from the Karolinska Directed Emotional Faces (KDEF) database^33^. Three different male-face pictures (CS1, CS2, and CS3) were presented in the center of a black screen (slides of 9,5 cm × 7 cm. Two of them (CS1 and CS2) expressed anger on their faces, and the other one (CS3) was neutral (no emotion). Each stimulus appeared 8 times (24 trials total) representing a moderate training intensity. In the trained groups, either CS1 or CS2 was associated with the unconditioned stimulus (US) in 75% of the trials in a counterbalanced manner. Hereinafter, we will call the aversive reinforced stimulus “CS+,” the unreinforced aversive stimulus “CSa” and the neutral stimulus “CSn”. An auditory stimulus (tone) with duration of 1, 5 s delivered through stereo headphones served as the US. All the CSs were presented for 6 s, and the US appeared 1, 5 s before CS offset. The interval between stimuli varied among 8 s, 10 s, and 12 s. The tone was generated by a TG/WN Tone-Noise Generator (Psychlab), digitally controlled with a mean of 98 db ± 4 db. The US was adjusted for each subject to be “unpleasant but not painful” (100 db was the maximum allowed for any subject). The no-training group received the same instruction and performed the same task in the absence of the tone-US. Threat conditioning was measured by the skin conductance response (SCR). The input device (Psychlab Precision Contact Instruments) has a sine excitation voltage (±0.5 V) of 50 Hz derived from the main frequency. The device was connected to two Ag/AgCl electrodes of 20 mm × 16 mm located in the intermediate phalanges of the non-dominant hand. The SCR produced by each CS was measured by taking the average baseline to the first peak within the 0,5–4,5 s window following the stimulus onset. A minimum response criterion of 0,002 microSiemens (µS) was used, and all the other responses were scored as zero^34,35^. Data were analyzed with MATLAB and Ledalab^36^. Only subjects who showed differential fear responses (CS+ SCR amplitude > CSa and CSn) were considered for analysis.

#### Stimuli Representation (aversiveness)

The *discrimination index* was determined using the mean difference in time (post- or pre-threat conditioning) between the aversive CS presented during threat conditioning [(_post_CS+_score_ − _pre_CS+_score_)/(_post_CSa_score_ − _pre_CSa _score_)]. The *generalization index* was estimated using the mean difference between all the aversive and neutral faces after threat conditioning divided by this difference before treatment [(_post_Aversiveented during threat conditioning_post_Neutral)/(_pre_Aversiveented during threat conditioning_pre_Neutral)].

#### Valuation (expected value)

To reduce complexity for statistical purposes, we calculated the difference between the negative and positive expected values between CS+ and CSa and retained only 2 expected values in total for each group [(Expected Value CS+_positive_) − (Expected Value CSa_positive_)] and [(Expected Value CS+_negative_) − (Expected Value CSa_negative_)]. An example of a negative event could be: “*How likely would it be for you to have problems at work with HIM?*” and then: “*How bad would it be for you to have problems at work with HIM?”* (always accompanied by the CS+ or CSa picture). In contrast, a positive situation could be: “*How likely would it be for HIM to take you to the airport?*” and then: “*How good would it be for HIM to take you to the airport?*”.

### Semantic fluency

Repeated words as well as those related to other categories were excluded from analysis.

#### Attentional bias

The probe position (left/right) and face location were counterbalanced. Subjects performed a total of 160 trials in two blocks of 80 (40 trials *per* condition). The mean RT from the dot probe task was estimated for each group *per* condition taking the difference between incongruent and congruent trials. Errors of commission (failure to detect the correct probe location) or omission (not responding) and responses less than 150 ms or greater than 2000 ms were not included in the analysis. Trials in which the RT was ±2 SD of the subjects’ mean for a specific condition were also excluded from analysis. The percentage of excluded responses was 1.9% for the Short-term evaluation groups and 2.2% for the Long-term evaluation Groups.

### Data analysis

The initial subjective assessments (STAI-T and BAI) was analyzed using an independent *t*-test between groups. Skin conductance response amplitudes in the threat conditioning were analyzed by means of a mixed ANOVA for repeated-measures with “group” as the between-subjects factor and “stimulus” (CS+, CSa and CSn) and “trial” (trials 1–8) as within-subjects factors. When the interaction was significant, simple effects were performed. When sphericity was not accomplished, Greenhouse−Geisser correction was applied. The main effect of threat conditioning on the target variables was analyzed using a two-way MANOVA with Group (trained vs not trained) and Time (short term vs long term evaluation) as independent factors and the negative valence, positive valence and cognitive systems as dependent variables, followed by separate Two-way ANOVA’s for each dependent variable and a discriminant function analysis. Then each Two-way ANOVA was followed by post hoc pairwise comparison using the Bonferroni correction for the main effects. Significant interactions were analyzed with simple effects and post hoc Tukey comparisons.

All raw data are presented in Table S1 (Supplemental Material).

### Code availability

All tasks were coded with MATLAB (MathWorks, Inc., Sherborn, MA, USA) and the Psychtoolbox module. The statistical analysis was calculated using *IBM SPSS Statistics* 22 and no custom code was generated for these experiments.

### Data availability

The datasets obtained in the current study are available from the corresponding author upon request.

## Electronic supplementary material

Supplementary Information
