# Supplementary material for: Effects of Threat Conditioning on the Negative Valanced Systems and Cognitive Systems
Source: Sci Rep. 2018 Jul 25;8:11221. doi: 10.1038/s41598-018-29603-3 (PMC6060145; doi:10.1038/s41598-018-29603-3)
Supplement: Supplementary file 1 — Supplementary Information [file 41598_2018_29603_MOESM1_ESM.docx]

**SUPPLEMENTAL MATERIAL:**

**EFFECTS OF THREAT CONDITIONING ON THE NEGATIVE VALANCED SYSTEMS AND COGNITIVE SYSTEMS**

**Rodrigo S. Fernández^a,1^, Soledad Picco^a,1^, Fernando Messore^a^ & María E. Pedreira^a*^**

**^a^** Laboratorio de Neurobiología de la Memoria, Departamento de Fisiología y Biología Molecular y Celular, IFIBYNE-CONICET.

**Table S1**

|  |  |  |  |  |
| --- | --- | --- | --- | --- |
|  | **Short-term evaluation** |  | **Long-term evaluation** |  |
|  | **no-TR Group** | **TR Group** | **no-TR Group** | **TR Group** |
| ***Subjective Assessement*** |  |  |  |  |
| **BAI** | 9,5 (1,3) | 10,4 (1,7) | 9,3 (1,6) | 9,7 (1,6) |
| **STAI-T** | 30,5 (1,4) | 32,1 (1,5) | 33,4 (2,2) | 32, 1 (1,6) |
|  |  |  |  |  |
| **Negative Valence and Positive Valence Systems** |  |  |  |  |
| **State Anxiety (STAI-S) Pre** | 31,3 (1,1) | 33,2 (1,3) | 33,4 (1,6) | 32,7 (1,6) |
| **State Anxiety (STAI-S) Post** | 28,5 (1,2) | 33,0 (1,0) | 30,8 (1,8) | 30,8 (1,7) |
|  |  |  |  |  |
| ***Stimuli Representation*** |  |  |  |  |
| ***Generalization Index*** |  |  |  |  |
| **Neutral Stimuli Pre** | 2,1 (0,3) | 2,0 (0,3) | 2,0 (0,2) | 2,4 (0,3) |
| **Neutral Stimuli Post** | 1,8 (0,3) | 1,7 (0,3) | 2,1 (0,3) | 1,9 (0,3) |
| **Aversive Stimuli Pre** | 4,5 (0,3) | 3,6 (0,3) | 3,7 (0,4) | 4,0 (0,4) |
| **Aversive Stimuli Post** | 4,6 (0,3) | 4,3 (0,3) | 4,1 (0,3) | 4,5 (0,4) |
|  |  |  |  |  |
| ***Discrimination Index*** |  |  |  |  |
| **CS+** | -0,1 (0,3) | 1,0 (0,3) | -0,1 (0,4) | 1,25 (0,3) |
| **CS-** | 0,7 (0,3) | 0,23 (0,4) | 0,4 (0,3) | 0,5 (0,3) |
|  |  |  |  |  |
| ***Valuation*** |  |  |  |  |
| **CS+ negative Expected Value** | 16,2 (1,1) | 19,5 (1,1) | 17,1 (1,5) | 16,8 (1,4) |
| **CS+ positive Expected Value** | 13,1 (1,0) | 10,7 (0,7) | 17,4 (1,2) | 13,5 (1,3) |
| **CS- negative Expected Value** | 18,2 (1,3) | 16,7 (1,2) | 20,0 (2,3) | 17,9 (1,6) |
| **CS- positive Expected Value** | 12,5 (0,9) | 11,1 (0,9) | 17,5 (1,3) | 12,1 (1,5) |
|  |  |  |  |  |
| **Cognitive Systems** |  |  |  |  |
| ***Semantic Fluency*** |  |  |  |  |
| **Neutral category** | 24,3 (1,5) | 26,2 (1,2) | 26,0 (2,1) | 24,9 (1,9) |
| **Aversive category** | 13,8 (1,0) | 13,0 (1,1) | 15,3 (1,1) | 14,5 (1,1) |
|  |  |  |  |  |
| ***Attentional Bias*** |  |  |  |  |
| **CS+ vs CSa Congruent** | 0,306 (0,01) | 0,294 (0,01) | 0,299 (0,01) | 0,298 (0,01) |
| **CS+ vs CSa Incongruent** | 0,309 (0,01) | 0,307 (0,01) | 0,303 (0,01) | 0,313 (0,01) |
| **CS+ vs CSn Congruent** | 0,315 (0,01) | 0,296 (0,01) | 0,316 (0,01) | 0,302 (0,01) |
| **CS+ vs CSn Incongruent** | 0,307 (0,01) | 0,305 (0,01) | 0,299 (0,01) | 0,311 (0,01) |
| **CSa vs CSn Congruent** | 0,304 (0,01) | 0,296 (0,01) | 0,307 (0,01) | 0,314 (0,01) |
| **CSa vs CSn Incongruent** | 0,303 (0,01) | 0,298 (0,01) | 0,305 (0,01) | 0,306 (0,01) |

**Table S2**

|  | **Rotated Factor Loadings** | |
| --- | --- | --- |
|  | **Negative valence systems** | **Cognitive Systems** |
| Attentional bias (CS+ vs CSa) | .757 |  |
| Attentional bias (CS+ vs CSn) | .661 |  |
| Stimuli representation (Generalization Index) | .650 |  |
|  |  | .660 |
| Stimuli representation (Discrimination Index) |  | .650 |
|  |  | .641 |
| State-anxiety |  |  |
| Valuation (negative expected value) |  |  |
|  |  |  |
